# Immunological Roles of Elevated Plasma Levels of Matricellular Proteins in Japanese Patients with Pulmonary Tuberculosis

**DOI:** 10.3390/ijms18010019

**Published:** 2016-12-22

**Authors:** Beata Shiratori, Jingge Zhao, Masao Okumura, Haorile Chagan-Yasutan, Hideki Yanai, Kazue Mizuno, Takashi Yoshiyama, Tadashi Idei, Yugo Ashino, Chie Nakajima, Yasuhiko Suzuki, Toshio Hattori

**Affiliations:** 1 Division of Disaster-Related Infectious Diseases, International Research Institute of Disaster Science, Tohoku University, 2-1 Seiryo-machi, Aoba-ku, Sendai, Miyagi 980-8575, Japan; beatabucekova@gmail.com (B.S.); zhaojingge1987@gmail.com (J.Z.); haorile@gmail.com (H.C.-Y.); ya82@yahoo.co.jp (Y.A.); 2 Department of Respiratory Medicine, Fukujuji Hospital, Japan Anti-Tuberculosis Association, 3-1-24 Matsuyama, Kiyose, Tokyo 204-8533, Japan; okumuram@fukujuji.org (M.O.); yoshiyamat@fukujuji.org (T.Y.); 3 Department of Clinical Laboratory, Fukujuji Hospital, Japan Anti-Tuberculosis Association, 3-1-2 4 Matsuyama, Kiyose, Tokyo 204-8533, Japan; yanaih@fukujuji.org (H.Y.); hiralin99@yahoo.co.jp (K.M.); ideit@fukujuji.org (T.I.); 4 Division of Global Epidemiology, Research Center for Zoonosis Control, Hokkaido University, North 20, West 10, Kita-ku, Sapporo, Hokkaido 001-0020, Japan; cnakajim@czc.hokudai.ac.jp (C.N.); suzuki@czc.hokudai.ac.jp (Y.S.); 5 Department of Health Science and Social Welfare, Kibi International University, 8 Igamachi, Takahashi 716-8508, Japan

**Keywords:** tuberculosis, osteopontin, galectin-9, CD44, Beijing genotype MTB

## Abstract

Elevated matricellular proteins (MCPs), including osteopontin (OPN) and galectin-9 (Gal-9), were observed in the plasma of patients with Manila-type tuberculosis (TB) previously. Here, we quantified plasma OPN, Gal-9, and soluble CD44 (sCD44) by enzyme-linked immunosorbent assay (ELISA), and another 29 cytokines by Luminex assay in 36 patients with pulmonary TB, six subjects with latent tuberculosis (LTBI), and 19 healthy controls (HCs) from Japan for a better understanding of the roles of MCPs in TB. All TB subjects showed positive results of enzyme-linked immunospot assays (ELISPOTs). Spoligotyping showed that 20 out of 36 *Mycobacterium tuberculosis* (MTB) strains belong to the Beijing type. The levels of OPN, Gal-9, and sCD44 were higher in TB (positivity of 61.1%, 66.7%, and 63.9%, respectively) than in the HCs. Positive correlations between OPN and Gal-9, between OPN and sCD44, and negative correlation between OPN and ESAT-6-ELISPOT response, between chest X-ray severity score of cavitary TB and ESAT-6-ELISPOT response were observed. Instead of OPN, Gal-9, and sCD44, cytokines G-CSF, GM-CSF, IFN-α, IFN-γ, IL-12p70, and IL-1RA levels were higher in Beijing MTB-infected patients. These findings suggest immunoregulatory, rather than inflammatory, effect of MCPs and can advance the understanding of the roles of MCPs in the context of TB pathology.

## 1. Introduction

In 2000, Bornstein et al. proposed that there is a family of secreted extracellular matrix (ECM) proteins termed “matricellular” proteins to highlight their influence on cell-matrix interactions [1]. Based on this definition, several proteins have now been identified as matricellular proteins (MCPs), including connective-tissue growth factors, galectins [2] and osteopontin (OPN) [3]. MCPs participate in wound repair, inflammation, and cancer progression by binding to their receptor [3]. The multitasking aspects of MCPs are derived from the different structural proteins, cell-surface receptors, proteases, and cytokines with which these proteins come into contact in the local environment of various tissues.

Among infectious diseases, *Mycobacterium tuberculosis* (MTB) infection remains a global public threat due to its ability to evade the host immune system by various mechanisms, including inhibition of phagolysosome fusion within phagocytes or induction of anti-inflammatory cytokine secretion [4]. Abnormal turnover of MCPs in the development of granulomas and cavities are the typical pulmonary manifestations of TB [5], in which chronic inflammation is activated, leading to tissue damage and subsequent tissue remodeling [6]. MCPs are expressed at low levels in normal adult tissues but are promptly up-regulated during tissue repair and remodeling processes [7]. In a previous study, we observed the expression of OPN and Gal-9 in TB granuloma [8]. We also confirmed the high level of plasma OPN in subjects with Manila genotype MTB from the Philippines [9] and in TB patients from Indonesia [8]. The intact form of OPN, also reported as full-length OPN (FL-OPN), is involved in the complex pathways of coagulation and fibrinolysis, where multiple sites of FL-OPN serve as a thrombin-cleaved target. During this process, the OPN fragments are produced. Among those fragments, proteolytic cleavage of FL-OPN by thrombin (between Arg168 and Ser169) generates a functional fragment of N-terminal thrombin-cleaved OPN (trOPN), which contains a cryptic binding site for integrins α9β1 and α4β1 that enhances the attachment of trOPN to integrins. Elevation of trOPN levels has been reported in the recovery phase of dengue virus infection [10].

Galectin-9 (Gal-9), a β-galactoside-binding MCP that induces apoptosis, chemoattraction, and necrosis, stimulates bactericidal activity in mouse TB models by binding to its receptor, T-cell immunoglobulin and mucin domain-containing molecule-3 (Tim3) [11,12]. Tim3-expressing T cells accumulate during chronic TB infection, produce less IL-2 and TNF but more IL-10, and are functionally exhausted. Such T-cell exhaustion impairs immunity and is detrimental to the outcome of MTB infection [13]. On the other hand, Gal-9 is reported to stimulate regulatory T cells and is produced by them in an autocrine manner, indicating that they have immunoregulatory functions [14]. Gal-9 and Tim-3 expression in CD4^+^ and CD8^+^ T cells increases during TB infection in humans compared to healthy individuals [15]. As a result, the recovery of T-cell function against MTB is associated with the blockage of TIM3 [16]. The associations of Gal-9 with the severity of the diseases were also found in dengue virus [17] and malaria infection [18], suggesting that manipulation of Gal-9 signals has an immunotherapeutic potential and may represent an alternative approach to improving immune responses to infections and/or vaccines [19]. Based on these findings, Gal-9 is proposed to be a soluble molecule responsible for an immune checkpoint [20].

CD44, a polymorphic transmembrane glycoprotein encoded by a single gene located on chromosome 11, one of OPN receptors, is involved in signaling and in regulating immune responses, and contributes to clinical manifestations [21]. Increased OPN and CD44 expression was reported in adult T-cell leukemia cells [22]. Meanwhile, CD44 glycosylation directly controls binding affinity of Gal-9 for fibrin and for immobilized fibrinogen and, therefore, participates in a wide variety of cell-cell or cell-matrix interactions, including tumor invasion and metastases [23]. CD44, along with CD25, is used to track early T-cell development in the thymus, and CD44 expression is an indicative marker for effector-memory T cells. Both functions involve a mechanism of CD44-regulated apoptosis resistance in T-cell subpopulations, namely T_h_1 cells [24]. On the other hand, the sCD44 level in TB patients has not been examined.

Interferon γ (IFN-γ)-producing TB antigen-specific CD4^+^ effector T cells and memory T cells can be monitored by an enzyme-linked immunospot assay (ELISPOT) [25], in which galectin-9–CD44 interaction enhances stability and function of adaptive regulatory T cells (Tregs), promoting Foxp3 expression and, therefore, suppressing effector T cell responses during infection [26]. Spoligotyping methods have been applied to identify the Beijing genotype of MTB that has been demonstrated as an independent risk factor of treatment failure [27]. In this study, results of various current diagnostic methods and clinical findings were also analyzed in view of the function of MCPs. Our results showed an important immunological role of elevated OPN, Gal-9, and sCD44 levels in MTB infection.

## 2. Results

### 2.1. Clinical Findings

The study includes 36 patients with active pulmonary TB, six LTBI patients, and 19 HCs. All HCs tested negative for T-SPOT.TB, and all TB patients tested positive for T-SPOT.TB. Laboratory data on the TB patients are summarized in Table 1. The majority of TB patients had low values of hemoglobin and hematocrit. Analysis of differential blood cell counts in TB patients showed frequent neutrophilia and lymphocytopenia. Plasma C-reactive protein (CRP) levels were above the reference range in 32 (88.8%) TB patients. Among the 36 patients with active pulmonary TB, 20 and 16 were infected with Beijing and non-Beijing type MTB, respectively. Moreover, 16 non-Beijing MTB isolates were found to be Latin American-Mediterranean-9 strain (one patient) and T2 strain (one patient); EAI2_Manila strain (two patients) and T3-OSA strain (two patients); a new type of strain (five patients) and T1 strain (five patients).

### 2.2. Luminex and ELISA

**Figure 1:**
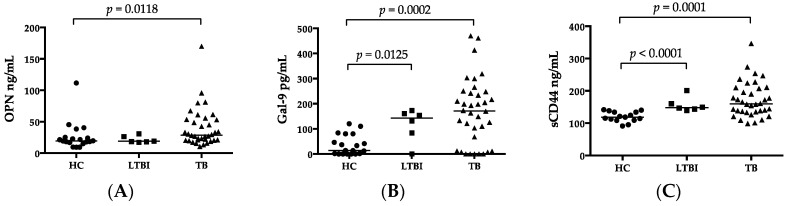

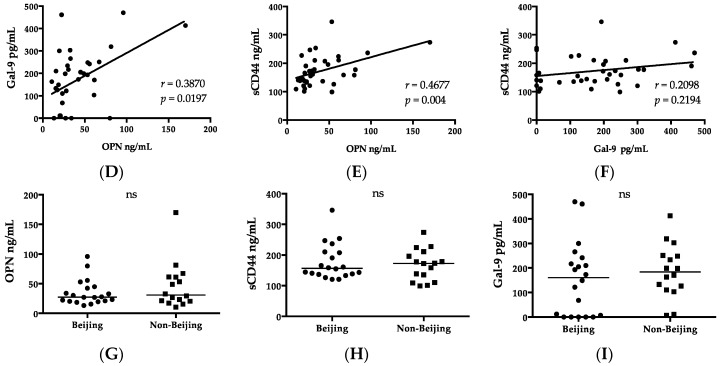
OPN, Gal-9 and sCD44 in groups under study. Comparison of plasma levels of OPN (**A**); Gal-9 (**B**); and sCD44 (**C**) among HC, LTBI, and TB patients. Correlations among OPN, Gal-9, and sCD44 in TB patients (**D**–**F**); comparison of OPN (**G**); Gal-9 (**H**); and sCD44 (**I**) between Beijing and non-Beijing genotype.

**Figure 2:**
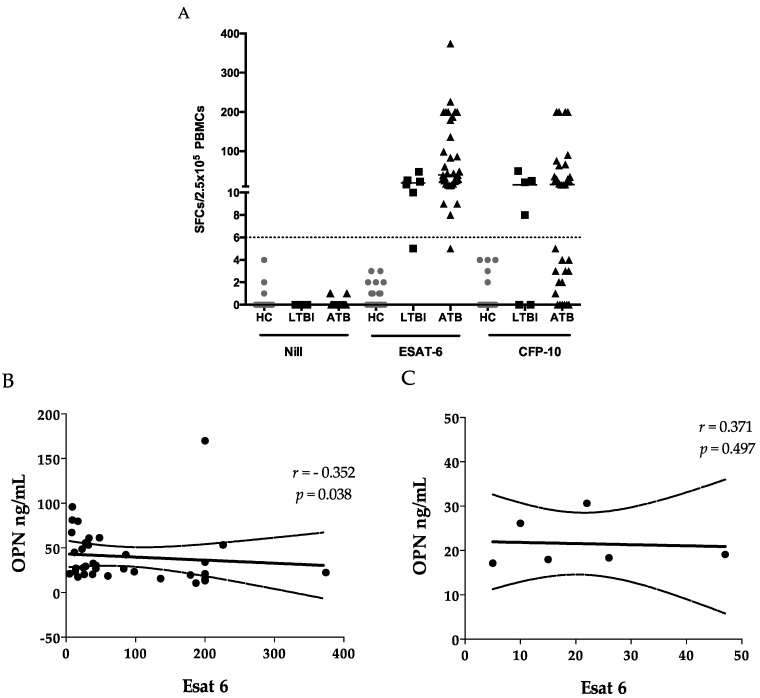
ELISPOTs reaction and OPN levels. ELISPOT assays are plotted as the number of specific PBMCs against the indicated stimulus (**A**); a negative correlation was observed between the ELISPOT for ESAT-6 and OPN in TB (**B**); and this correlation was not seen in the LTBI group (**C**).

**Figure 3:**
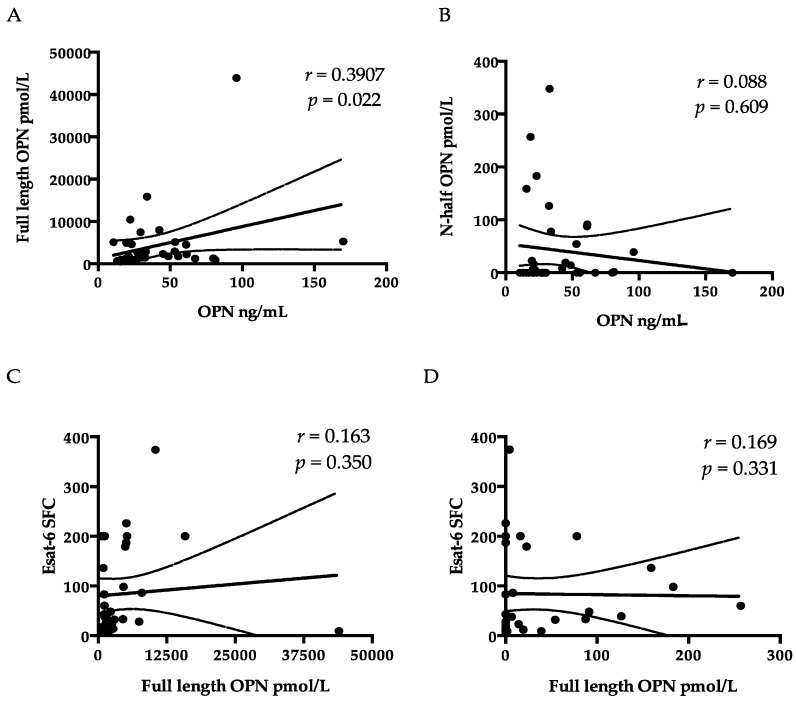
OPN, full-length OPN, n-half OPN, and ESAT-6 SFC count in TB patients. A comparison of plasma levels of OPN with full-length OPN (**A**) and with n-half OPN (**B**); and a comparison of ESAT-6 ELISPOT SFC counts with full-length OPN (**C**) and with n-half OPN (**D**).

Luminex assay was applied to determine the levels of pro-inflammatory markers that are important in cell signaling and promote systemic inflammation. The results showed high levels of IFN-γ, IL-8, IP-10, and TNF-α in the TB group compared to HCs (Table 2). Plasma levels of other 25 biomarkers were below the measurable levels or did not show differences among the groups. Plasma OPN, Gal-9, and sCD44 were significantly higher in TB patients than in HCs. Plasma Gal-9 and sCD44 concentrations were higher in patients with LTBI than in HCs. In contrast, despite low levels of OPN in both HCs and LTBI, there was no significant difference between patients with TB and patients with LTBI (Figure 1A–C). Unlike OPN, the FL-OPN and trOPN levels in the TB group did not differ from those in the HCs (Table 2). Spearman′s correlation analysis revealed a significant correlation between OPN and Gal-9, and between sCD44 and OPN, but not between Gal-9 and sCD44 in TB patients (Figure 2D–F). In TB patients, the levels of both OPN and sCD44 were associated with lymphocytopenia and the levels of CRP, IL-8, IP-10, and OPN significantly correlated with neutrophils, whereas sCD44 correlated with white blood cell counts and TNF-α, but such a correlation was not observed in HCs. In spite of the significant correlation between OPN and FL-OPN in the TB patients, OPN did not correlate with trOPN (Figure 3A,B). Although Gal-9 did not show a correlation with either OPN or sCD44 (Table 3), there was a correlation of Gal-9 with alanine aminotransferase (ALT) and creatinine. Plasma OPN, Gal-9, and sCD44 did not discriminate between patients with Beijing and non-Beijing MTB (Figure 1G–I).

Of the 29 cytokine/chemokine indicators examined in each group, 15 indicators (IL-1α, IL-1β, IL-2, IL-3, IL-4, IL-5, IL-6, IL-7, IL-10, IL-12p40, IL-13, IL-15, IL-17A, MIP-1α, and TNF-β) were excluded from statistical analyses because their median levels were below the detection level. EGF, VEGF, MCP-1, MIP-1β, eotaxin, G-CSF, GM-CSF, IFN-α2, IL-12p70, and IL-1RA did not differ among the groups. OPN, Gal-9, and sCD44 differed between groups. FL-OPN and trOPN did not differ among the groups.

### 2.3. ELISPOTS

All HCs tested negative for ESAT-6 or CFP-10 because the SFC (cutoff = 6) was lower than the cutoff; none of them showed total SFC ≥ 8 in ESAT-6 and CFP-10 assays. In the TB group, one sample was negative for ESAT-6 (SFC = 5) and CFP-10 (SFC = 3); the total SFC was no less than 8, and the result was considered positive. In the LTBI group, one sample with negative Esat-6 (SFC = 5) and CFP-10 (SFC = 0) was considered indeterminate because total SFC was less than 8. Therefore, the sensitivity of ELISPOTs for TB and LTBI was 100% and 83.3%, respectively (Figure 1A). There was a negative correlation between ESAT-6 SFC and OPN levels (Figure 1B) in TB but not in LTBI (Figure 1C). Nonetheless, ESAT-6 did not correlate with either FL-OPN or trOPN (Figure 3C,D), nor was a correlation detected between OPN levels and CFP10. Moreover, none of 19 markers tested by Luminex showed any correlation with either ESAT-6 or CFP-10 responses.

### 2.4. Sensitivity and Specificity

**Figure 4:**
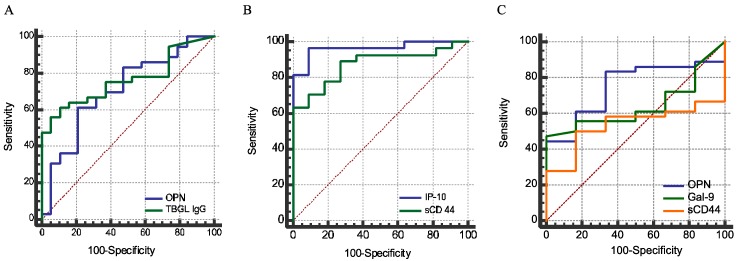
Sensitivity and specificity analysis for OPN, anti-TBGL IgG, IP-10, and sCD44. Comparison between ATB and HCs (**A**,**B**), no significant difference between the areas under curve (AUC) of OPN and anti-TBGL IgG, IP-10, or sCD44, *p* > 0.05. Strong discriminatory power of IP-10 and sCD44; *p* < 0.0001 for both (**A**). Comparison between ATB and LTBI, no significant discriminatory power of OPN, Gal-9, and sCD44 in LTBI and ATB (*p* > 0.05) due to the inadequacy of LTBI subjects (**C**). No significant difference between the AUC of OPN and Gal-9, between Gal-9 and sCD44, but difference between OPN and sCD44 (*p* = 0.014) (**C**).

ROC analysis revealed that OPN, Gal-9, sCD44, IP-10, and anti-TBGL IgG could discriminate active TB (ATB) from HC, in spite of varied discriminatory power indicated by an AUC comparison. IP-10 (92.6% sensitivity and 93.3% specificity) was more effective than OPN (*p* < 0.001) and anti-TBGL IgG (*p* < 0.05). sCD44 (63.9% sensitivity and 100% specificity) was more effective than OPN (Table 4). Of note, in spite of greater discriminatory power of IP-10 and sCD44, there was no significant difference between these two tests (Figure 4B), nor was a difference found between OPN and anti-TBGL assays (Figure 4A). No valid discrimination was found between ATB and LTBI in terms of the MCPs due to small number of LTBI subjects (Figure 4C).

### 2.5. Clinical Biomarkers, Chest X-rays, and Genotype

OPN and sCD44 showed positive correlations with inflammatory markers and a negative correlation with lymphocyte counts (*p* < 0.001). On the contrary, Gal-9 levels did not show any correlation with inflammatory and hematological markers, but correlated positively with ALT and creatinine (Table 3). Analysis of patients′ chest radiographs showed cavity formation in 12 (33.3%) patients. Patients with lung cavities had significantly lower levels of hemoglobin and hematocrit and a higher differential number of monocytes and higher CRP, anti-TBGL IgG, and IP-10 levels (Table 5). In contrast, the levels of OPN, sCD44, and Gal-9 were not significantly higher in cavity-positive ATB subjects.

The CXR score correlated with a higher proportion or higher number of monocytes and IL-12p70. The CXR score in cavity-positive and cavity-negative TB patients was analyzed further. In the former subgroup, ESAT-6 SFC showed an inverse correlation with CXR (Table 6). In the latter subgroup, IL-12p70 correlated with CXR (Table 6). No significant differences in plasma OPN, Gal-9, or sCD44 were observed between subjects with Beijing and non-Beijing MTB (Figure 1G–I). Nonetheless, higher G-CSF, GM-CSF, IFN-α, IFN-γ, IL-12p70, and IL-1RA concentrations were detected in Beijing MTB subjects (Table 7). Among these six makers, levels of IFN-α, IFN-γ, and IL-1RA were higher in Beijing MTB subjects compared to the HCs (Table 7, *Kruskal-Wallis* test, *p* < 0.05).

## 3. Discussion

It is known that cells producing IFN-γ after stimulation with ESAT-6 and CFP-10 are CD4^+^ effector memory cells in both HIV-infected [28] and uninfected subjects [29]. A very low proportion of MTB-specific effector T cells is found in the blood compared with the infected tissue, indicating the differences in the cellular immune response and regulatory mechanisms between focal sites and systemic levels [30]. OPN is a multifunctional phosphorylated glycoprotein that is synthesized by a variety of immune and non-immune cells, and it participates in the balance between the T_h_1 and T_h_2 responses and in granulomatous reactions [31,32]. A negative correlation was observed between OPN and ESAT-6 ELISPOTs (Figure 1B), in addition to the negative correlation of OPN with lymphocyte counts (Table 3), which could be explained by increased migration of lymphocytes toward the lesion in response to OPN signaling [33]. OPN-induced T-cell migration may initiate suppression of hyperinflammation [33] and prevent the contact between peripheral lymphocytes and MTB bacilli, to the extent of compartmentalization of MTB bacteria and lower risk of dissemination [34,35]. Since OPN showed a correlation with sCD44, one of the memory T-cell markers [36], it may also reflect the activation of memory T cells in the lesion. Of note, we did not observe a correlation between OPN and a CFP-10 cellular response, in support of the discrepancies between results of ESAT-6 and CFP-10 assays [37]. Nevertheless, the component of secreted OPN responsible for summoning T-cell immigration is still unknown. Therefore, we tested the correlation among ESAT-6 SFC, FL-OPN, and trOPN, which did not show a statistical association with one another. It is more than obvious that the FL-OPN level exceeds the trOPN level, in addition to a correlation between OPN and FL-OPN, suggesting that FL-OPN is responsible as one of components of OPN. Another culprit may be MMP-cleaved OPN [32]. Nevertheless, these components were not demonstrated in this study.

OPN levels are higher in patients with extensive TB/HIV coinfection than in patients with a single disease of TB or HIV [38]. HIV has been proposed to infect memory T cells preferentially, and efficient transfer of the R5 virus to effector memory T cells has also been observed [39,40]. We have reported the increased amount of OPN in an AIDS-TB case, though this patient showed lymphadenopathy and did not have granuloma of the lungs [41]. Probably, OPN was synthesized in activated lymph nodes and immune cells in this patient, and we have reported that macrophages are the main producer in the lymph nodes of adult T-cell leukemia patients [21]. In this particular case of AIDS/TB, OPN did not decrease after antiretroviral therapy despite the fall of the viral load [41], and OPN was retained as a component of the immune reconstitution (IRIS) that takes place during antiretroviral therapy [42]. These data suggest that OPN could be synthesized in response to both TB and HIV infection and serve as a marker of complex disease activity such as IRIS.

Gal-9 appeared to reflect disease severity as reported for other diseases, such as malaria and dengue, because of its association with ALT and creatinine [17,18] (Table 3). The levels of ALT and creatinine are indicators of systemic severity of TB infection [43]. The association of these molecules with Gal-9 in TB cases supports the idea that Gal-9 could either influence the outcome of MTB infection or indicate the state of disease [44]. Like many immunological pathways, the Gal-9 pathway functions via binding of Gal-9 to its receptor TIM3 prior to the initiation of the MCP-mediated signaling, before regulation of intracellular antimicrobial processes and of long-term immunological memory, as well as physiological homeostasis [45]. In vitro TIM3 blockade—in co-culture experiments with MTB-infected macrophages from TB patients with or without HIV co-infection—promotes bacterial killing and enhances IL-1β secretion by infected cells, as well as the IFN-γ release by T cells [12]. Therefore, the interaction of Gal-9 and TIM3 serves as an immune checkpoint rather than leading to inflammation [20]. Regimens incorporating therapeutics targeting such immune checkpoints are urgently needed to improve the clinical management of multidrug-resistant TB (MDR-TB) when the TB drug options are diminished for patients with MDR-TB infection [46]. Drugs enhancing T-cell activity, depleting Treg cells, and inhibiting the immune checkpoint have been reported, in agreement with the potential of targeting of the Gal-9–TIM3 interaction.

In this study, for the first time, we reported a high plasma concentration of sCD44 in ATB compared to HCs, in the sense of discriminatory power comparable to that of IP-10 (Figure 4B, *p* > 0.05). The discriminatory power of sCD44 is higher than that of OPN. Nonetheless, like OPN and Gal-9, sCD44 is related to immune regulation. Without the recruitment of other non-MTB inflammatory diseases in this study, it is implausible to make a conclusion about the reliability of these markers for TB diagnosis. IP-10, a chemokine secreted from cells stimulated with interferon and lipopolysaccharides, is a chemoattractant for activated T cells [47]. IP-10 concentrations correlated with the plasma OPN level (Table 3). A similar finding has also been reported, except that decreased IP-10 and OPN levels were reported as markers of negative conversion in sputum smears. Nevertheless, only IP-10 correlates with CRP and inflammation [48]. In our study, significantly higher levels of plasma IP-10 in cavitary TB patients (Table 5), and the correlation between IP-10 and CXR score (Table 6) are in agreement with IP-10’s role as an inflammation inducer. In addition to an inflammatory marker of TB [49], serum IP-10 also increased in chronic hepatitis C [50] and autoimmune diseases [51].

Beijing genotype MTB has been the most prevalent in East Asia [52] because of its virulence and resistance to drugs and BCG vaccination. Treatment failure and relapse have also been found to be associated with Beijing genotype MTB [53]. On the other hand, other researchers, and our group, have reported that the rates of MDR-MTB among Beijing and non-Beijing family strains are not statistically significantly different in Beijing MTB-predominant regions [27,54]. Highly intense inflammation of Beijing genotype MTB infection may be detected by assaying inflammatory cytokines. Nevertheless, we did not detect differences in IP-10, sCD44, OPN, or Gal-9 concentrations between Beijing and non-Beijing TB infections; plasma concentrations of other cytokines, including G-CSF, GM-CSF, IFN-α, IFN-γ, IL-12p70, and IL-1RA, were found to be higher in Beijing MTB-infected subjects than in non-Beijing MTB-infected subjects. An increased level of IL-12p70 was found to be associated with a high CXR sore, in support of the more severe lung damage in Beijing MTB infection compared to non-Beijing MTB infection [55]. G-CSF and GM-CSF also play a role in the regulation of macrophages and dendritic cells to facilitate granuloma in the development of a cavity [56]. Therefore, a high percentage of patients with a cavity were observed in the Beijing MTB group (40%) compared to the non-Beijing MTB group (25%) in this study. Unlike those cytokines, MCPs are involved in not only inflammation, but also immune regulation, and their concentrations were not affected by the genotype of MTB in this study.

## 4. Materials and Methods

### 4.1. Study Subjects

The study was conducted at Double-Barred Hospital, Tokyo, Japan, and Tohoku University, Sendai, Japan, between May 2014 and 2015. The study protocol was approved by the Ethics Committee of Fukujuji Hospital, Japan Anti-Tuberculosis Association and Graduate School of Medicine (NO. 2014-1-122, January 2014), Tohoku University. Written informed consent was obtained from all the enrolled subjects. All of the procedures were conducted in accordance with the Declaration of Helsinki.

Patients with culture-confirmed TB diagnosis according to WHO guidelines [57] were included in the active TB group (*n* = 36); none of these patients had taken anti-TB medication. Healthy control subjects (HCs; *n* = 19) had no TB-related symptoms, and exhibited negative T-SPOT.TB (Oxford Immunotec, Oxford, UK) results. Subjects who showed positive T-SPOT.TB results or were positive in Interferon-γ release assays (IGRAs) and without TB clinical manifestations were categorized as patients with latent tuberculosis (LTBI; *n* = 6). Exclusion criteria were as follows: impossibility to obtain informed consent, cancer, and human immunodeficiency virus infection.

Each subject donated a 7-mL heparinized and 7-mL EDTA-treated peripheral-blood sample. The EDTA-blood samples were centrifuged within 30 min of collection, and plasma was stored at −80 °C until further analyses. Heparinized blood samples were sent from Double-Barred Cross Hospital and delivered to Tohoku University by a courier service within 24 h. All laboratory data were obtained from patients’ medical records and at the point of sample collection.

The TB patients were categorized into cavity-positive or cavity-negative in accordance with the presence or absence of cavities on chest X-ray images. A scoring method was used on the basis of the affected lung area and the presence of a cavity in order to conduct the comparison of TB lung lesion severity. The score was calculated as the percentage of lung affected plus 40 if cavitation was present [58].

### 4.2. Spoligotyping

To differentiate Beijing and non-Beijing genotypes of MTB, spoligotypes of clinical MTB isolates were determined as described previously [19]. Acid-fast bacilli (AFB) smear staining and Ogawa medium culture were conducted to confirm the MTB infection. To identify the most prevalent genotype, DNA samples were isolated from the colonies in culture. One colony was picked and resuspended in 0.5 mL of Tris-EDTA. The mixture was subjected to a 95 °C boil-and-cool cycle for decontamination before processing for spoligotyping. Briefly, the DR region was amplified with a primer pair, and the polymerase chain reaction (PCR) products were hybridized to a set of 43 spacer-specific oligonucleotide probes, which were covalently bound to membranes. The spoligo-international type was determined by comparing spoligotypes with the international spoligotyping database [20].

### 4.3. ELISPOTs

Peripheral blood mononuclear cells (PBMCs) were isolated from heparinized blood samples over Ficoll-Paque Plus (GE Healthcare Bio-Sciences AB, Uppsala, Sweden) and resuspended in the AIM V medium (Gibco, Grand Island, NE, USA) at the concentration of 2.5 × 10^5^ per 100 µL. *M. tuberculosis* infection was determined using T-SPOT.TB (Oxford Immunotec, Oxford, UK), according to the manufacturer’s recommendation. A test result was considered reliable if the spot-forming cell (SFC) number in the positive-control well was >20, and in the negative control well was <10. Positive results were scored as positive if the SFC number of either ESAT-6 or CFP-10 well was >6. If the total number of SFCs of ESAT-6 and CFP-10 was ≤8, the test result was considered indeterminate. Spots were counted with an automated Immunospot Analyzer, CTL (Cellular Technologies, Cleveland, OH, USA).

### 4.4. ELISA

Plasma concentrations of OPN were determined using the Human Osteopontin DuoSet ELISA Development System Kit (R and D Systems, Minneapolis, MN, USA) [21]. In this ELISA kit, the proprietary capture monoclonal antibody and the detection polyclonal antibodies were both raised against recombinant human OPN (NS0-derived, amino acids Ile17-Asn300). To determine the full-length OPN and trOPN, two separate ELISA kits (IBL, Gunma, Japan) were used. In the FL-OPN kit, a polyclonal rabbit antibody (O-17) specific to the N terminus of OPN (Ile17-Gln31, accession # NP_000573.1) was used as a capture antibody, and a mouse monoclonal antibody (10A16) raised against synthetic peptides corresponding to the internal sequence of human OPN (Lys166-Glu187) served as a detector antibody. Therefore, this kit does not allow us to detect trOPN. Meanwhile, the trOPN ELISA assay was performed using an anti-trOPN monoclonal antibody (34E3) as the capture antibody, and the O-17 antibody as the detection antibody. This capture antibody specifically reacts to the epitope Ser162–Arg168 exposed by thrombin and does not react with matrix metalloproteinase 3 or 7 (MMP-3 or -7)-cleaved N-terminal trOPN [10,59]. Gal-9 was quantified using a human Gal-9 ELISA kit (Galpharma Co., Ltd., Takamatsu, Japan), as described previously [17]. The concentration of soluble CD44 was measured by means of the Human CD44 ELISA kit (Abcam, Cambridge, MA, USA), as described previously [21]. Samples, reagents, and buffers were prepared according to the manufacturers′ manuals.

### 4.5. Luminex Assays

Twenty-nine cytokine and chemokine species, including epidermal growth factor (EGF), eotaxin, granulocyte macrophage-colony stimulating factor (GM-CSF), G-CSF, interferon-alpha2 (IFN-α), IFN-γ, interleukin 1 alpha (IL-1α), IL-1β, IL-1 receptor antagonist (IL-1RA), IL-2, IL-3, IL-4, IL-5, IL-6, IL-7, IL-8, IL-10, IL-12p40, IL-12p70, IL-13, IL-15, IL-17a, IFN-γ-inducible protein-10 (IP-10), monocyte chemotactic protein 1 (MCP-1), macrophage-inducible protein 1α (MIP-1α), MIP-1β, tumor necrosis factor α (TNF-α), TNF-β, and vascular endothelial growth factor (VEGF), in plasma were measured using a commercially available kit (Milliplex Human Cytokine and Chemokine multiplex assay kit, Merck Millipore, Billerica, MA, USA) by Luminex methods, as reported previously [17]. The assay was performed according to manufacturer′s instructions and the concentrations of cytokines/chemokines were calculated by comparing the assay readings with a five-parameter logistic standard curve on a Bioplex-200 instrument (Bio-Rad, Hercules, CA, USA). All of the results were expressed in pg/mL.

### 4.6. Data Analyses

Data are expressed as the median and range. Significance of differences for more than two groups was tested by the Kruskal–Wallis analysis. Significance of differences between two groups was tested by the Mann-Whitney *U* analysis. Correlations were determined using Spearman′s nonparametric test. These analyses were carried out in the GraphPad Prism 6 software (GraphPad, San Diego, CA, USA). Furthermore, receiver operating characteristic (ROC) curves were constructed to study the diagnostic utility of OPN, Gal-9, sCD44, and IP-10. The area under curve (AUC) and cutoff analyses were conducted by means of the MedCalc statistical software Version 16.8.4 (Ostend, Belgium). A difference was assumed to be significant at *p* < 0.05.

## 5. Conclusions

In conclusion, this study showed that the levels of OPN, Gal-9, sCD44, and IP-10 could help to understand the immune network of MCPs in TB, in addition to their diagnostic value in MTB infections, especially in the presence of a lung cavity. Higher plasma concentration of OPN in association with a low-ESAT-6-ELISPOT-response could help to understand the immunopathogenesis of TB. A high level of sCD44 and Gal-9 in MTB infection could also predict MTB-related inflammation and clinical severity.

## Figures and Tables

**Table 1:** Characteristics of HC, LTBI, and TB individuals.

Parameter	Ref.	HC (*n* = 19)	LTBI (*n* = 6)	TB (*n* = 36)	*p* Value
Antropometric data					
Age: year; median (range)		34 (19–67)	63 (36–71)	59.5 (19–86)	0.007
Gender: male; *n* (%)		12 (63)	5 (83)	28 (78)	0.43
Laboratory findings: median (range)					
RBC (10^6^/μL)	Male 4.5–5.5; Female 4.0–5.0	na	4.65 (4.35–5.12)	4.44 (2.72–5.57)	0.443
Hemoglobin (g/dL)	14–18	na	14.6 (14.2–15.3)	13.1 (8.9–17.5)	0.064
Hematocrit (%)	40–48	na	43.6 (40.9–46.0)	39.65 (25.5–51.9)	0.059
WBC (10^3^/μL)	4.5–11	na	7.64 (5.34–9.96)	7.33 (3.84–16.22)	0.945
Neutrophil (%)	38–80	na	57.35(51.9–75.7)	75.2 (57.0–89.6)	0.014
Lymphocyte (%)	15–40	na	33.1 (18.2–39.6)	14.1 (2.8–34.8)	0.007
Monocyte (%)	4–7	na	6 (4.9–7.7)	6.9 (3.4–11.2)	0.035
Eosinophil (%)	0–8	na	1.1 (0.4–4.1)	1.4 (0–11.4)	0.902
Platelet (10^3^/μL)	140–390	na	267 (203–280)	305 (125–564)	0.228
CRP (mg/dL)	0–0.3	na	0.055 (0.04–0.07)	6 (0.04–21.5)	0.002
Genotype (Beijing strain MTB %)	73.0, year 2010, Japan	na	na	55.6	na
ALT (Units)	4–37	na	20.5 (11–29)	15 (8–103)	na
Creatinine (mg/dL)	0.5–1.5	na	0.74 (0.42–11)	0.71 (0.57–0.86)	na

Age differences among the groups were analyzed by the *Kruskal-Wallis* test, gender differences by the *Chi-square* test, and laboratory findings by the *Mann-Whitney* test; *p* < 0.05 means a significant difference; na: not applicable.

**Table 2:** Biomarker levels measured by Luminex assay and ELISA in HCs and TB patients.

Biomarker	HC	TB	*p* Value
IFNγ (pg/mL)	4.46 (0.42–19.38)	8.13 (2.12–41)	0.0065
IL-8 (pg/mL)	1.585 (0.38–7.52)	7.26 (0.6–31.07)	<0.0001
IP-10 (pg/mL)	235.8 (132.2–472.8)	864.6 (219.3–3051)	<0.0001
TNFα (pg/mL)	3.88 (2.83–8.34)	10.11 (2.19–24.83)	<0.0001
OPN (ng/mL)	19.63 (9.31–111.64)	28.62 (10.59–170)	0.012
Gal-9 (ng/mL)	14.0 (0–120)	171.5 (0–470)	0.0002
sCD44 (ng/mL)	118.57 (91.02–141.41)	159.66 (98.89–346.2)	<0.0001
FL-OPN (nmol/mL)	5.20 (2.83–14.45)	1.75 (0.52–43.91)	>0.05
rtOPN (pmol/mL)	0 (0–26.73)	0.87 (0–347.95)	>0.05

**Table 3:** Correlations of OPN, Gal-9, and sCD44 with other laboratory parameters and biomarkers in TB patients.

Measurements	OPN	sCD44	Gal-9
	r (*P*)	r (*P*)	r (*P*)
WBC (10^3^/μL)	Ns	0.388 (0.019)	Ns
Neutrophil (%)	0.517 (<0.0001)	Ns	Ns
Lymphocyte (%)	−0.569 (<0.0001)	−0.558 (<0.0001)	Ns
CRP (mg/dL)	0.757 (<0.0001)	0.534 (0.001)	Ns
IL-8 (pg/mL)	0.474 (0.013)	0.524 (0.005)	Ns
IP-10 (pg/mL)	0.420 (0.029)	0.542 (0.003)	Ns
TNFα	Ns	0.446 (0.020)	Ns
ALT	Ns	Ns	0.375 (0.024)
Cre	Ns	Ns	0.377 (0.023)

Ns: not significant.

**Table 4:** Sensitivity and specificity.

Analytes	Youden Index	Cutoff	Sensitivity (%)	Specificity (%)	AUC	Comparison		
OPN	0.4006	25.1	61.1	79	0.706	***a***	***b***	
Gal-9	0.6667	120	66.7	100	0.798			
sCD44	0.6389	141.4	63.9	100	0.846	***a***		
IP-10	0.8593	400.3	92.6	93.3	0.965		***b***	***a***
Anti-TBGL IgG	0.5058	1.6	61.1	89.4	0.762			***a***

A cutoff was calculated based on TB and HC data from the Youden index. The discriminatory power of each test was evaluated by the area under curve (AUC) comparison. ***a***, ***b***, a significant difference between the AUCs of the indicated tests; ***a***, *p* < 0.05, ***b***, *p* < 0.001.

**Table 5:** Comparison of laboratory findings between TB patients with and without cavity lung formation.

Parameter	Normal Value (Range)	Cavity (−) (*n* = 24)	Cavity (+) (*n* = 12)	*p* Value
Hemoglobin (g/dL)	14–18	13.8 (8.9–17.5)	12.4 (9.9–14.7)	0.0454
Hematocrit (%)	40–48	42 (25.5–51.9)	37.5 (28.4–41.4)	0.0208
Monocyte (%)	4–7	6.2 (3.4–10)	8.3 (4.9–10.2)	0.0054
CRP (mg/dL)	0–0.3	3.27 (0.04–21.5)	8.67 (0.08–17.97)	0.0478
IP-10 (pg/mL)	132.2–472.8	751.5 (219.3–2735)	1570 (369.7–3051)	0.0408
Anti-TBGL IgG (U/mL)	<2	13.3 (0.1–62.6)	1.11 (0–72.6)	0.0123
CXR Score	0	92.46 (82.4–100)	131.84 (104.34–140)	0.0001

**Table 6:** CXR score and biomarkers in TB patients.

Parameter	CXR Scorer (*p*)	CXR Scorer (*p*)	
		Cavity (+)	Cavity (−)
Monocytes (%)	0.502 (0.003)	Ns	Ns
OPN	Ns	Ns	Ns
sCD44	Ns	Ns	Ns
IP-10	0.452 (0.027)	Ns	Ns
Gal-9	Ns	Ns	Ns
IL-12p70	0.517 (0.01)	Ns	0.574 (0.032)
ESAT-6 SFC	Ns	−0.6185 (0.0425)	Ns

Ns: no significant.

**Table 7:** Laboratory and biomarker values in patients infected by Beijing or non-Beijing strains of *Mycobacterium tuberculosis* (MTB).

Parameter	HC	Beijing Type (*n* = 20)	Non-Beijing Type (*n* = 16)	*p* Value
Laboratory findings				
RBC (10^6^/μL)	na.	4.28 (2.72–5.16)	4.64 (3.86–5.57)	0.0465
Total protein (g/dL)	na.	7.05 (5.41–8.03)	7.69 (6.54–8.14)	0.0323
Biomarker (pg/mL)				
G-CSF	48.48 (12.19–90.49)	56.94 (21.69–106.36)	38.12 (14.06–73.07)	0.0388
GM-CSF	4.24 (0.44–18.98)	12.29 (0.83–48.8)	5.84 (0.44–14.6)	0.0300
IFNα	11.07 (0–56.49)	32.87 (1.67–60.81) ^a^	7.53 (0–40.97)	0.0034
IFNγ	4.46 (0.42–19.38)	16.02 (3.09–41) ^a^	4.71 (2.12–17.84)	0.0141
IL-12p70	2.74 (1.35–15.06)	8.22 (1.85–29.77)	2.62 (0.03–7.13)	0.0007
IL-1RA	17.79 (0–81.26)	51.96 (8.56–217.37) ^a^	14.02 (0–78.44)	0.0095
IP-10	235.78 (132.2–472.75)	877.12 (219.29–2814.69) ^a^	864.57 (369.67–3051.48) ^b^	>0.05
Cavity *n* (%)	0	8 (40)	4 (25)	>0.05
CXR score	0	102.23 (82.4–140)	109.74 (82.77–140)	>0.05

Differences among the groups in terms of laboratory biomarkers were analyzed by the *Kruskal-Wallis* test; *p* < 0.05 indicates a significant difference between Beijing and non-Beijing MTB groups; ^a^ indicates a significant difference between groups Beijing MTB and HC, *p* < 0.05; ^b^ indicates a significant difference between groups non-Beijing MTB and HC; *p* < 0.05. na., no applicable.

## References

[1] Bornstein P., Sage E.H. (2002). Matricellular proteins: Extracellular modulators of cell function. Curr. Opin. Cell Biol..

[2] Elola M.T., Wolfenstein-Todel C., Troncoso M.F., Vasta G.R., Rabinovich G.A. (2007). Galectins: Matricellular glycan-binding proteins linking cell adhesion, migration, and survival. Cell. Mol. Life Sci. CMLS.

[3] Murphy-Ullrich J.E., Sage E.H. (2014). Revisiting the matricellular concept. Matrix Biol. J. Int. Soc. Matrix Biol..

[4] Deretic V., Singh S., Master S., Harris J., Roberts E., Kyei G., Davis A., de Haro S., Naylor J., Lee H.H. (2006). *Mycobacterium tuberculosis* inhibition of phagolysosome biogenesis and autophagy as a host defence mechanism. Cell. Microbiol..

[5] Elkington P.T., Emerson J.E., Lopez-Pascua L.D., O'Kane C.M., Horncastle D.E., Boyle J.J., Friedland J.S. (2005). *Mycobacterium tuberculosis* up-regulates matrix metalloproteinase-1 secretion from human airway epithelial cells via a p38 MAPK switch. J. Immunol..

[6] Dheda K., Booth H., Huggett J.F., Johnson M.A., Zumla A., Rook G.A. (2005). Lung remodeling in pulmonary tuberculosis. J. Infect. Dis..

[7] Cox T.R., Erler J.T. (2011). Remodeling and homeostasis of the extracellular matrix: Implications for fibrotic diseases and cancer. Dis. Models Mech..

[8] Hasibuan F.M., Shiratori B., Senoputra M.A., Chagan-Yasutan H., Koesoemadinata R.C., Apriani L., Takahashi Y., Niki T., Alisjahbana B., Hattori T. (2015). Evaluation of matricellular proteins in systemic and local immune response to *Mycobacterium tuberculosis* infection. Microbiol. Immunol..

[9] Shiratori B., Leano S., Nakajima C., Chagan-Yasutan H., Niki T., Ashino Y., Suzuki Y., Telan E., Hattori T. (2014). Elevated OPN, IP-10, and neutrophilia in loop-mediated isothermal amplification confirmed tuberculosis patients. Mediat. Inflamm..

[10] Chagan-Yasutan H., Lacuesta T.L., Ndhlovu L.C., Oguma S., Leano P.S.A., Telan E.F.O., Kubo T., Morita K., Uede T., Dimaano E.M. (2014). Elevated levels of full-length and thrombin-cleaved osteopontin during acute dengue virus infection are associated with coagulation abnormalities. Thromb. Res..

[11] Jayaraman P., Sada-Ovalle I., Beladi S., Anderson A.C., Dardalhon V., Hotta C., Kuchroo V.K., Behar S.M. (2010). Tim3 binding to galectin-9 stimulates antimicrobial immunity. J. Exp. Med..

[12] Sada-Ovalle I., Chavez-Galan L., Torre-Bouscoulet L., Nava-Gamino L., Barrera L., Jayaraman P., Torres-Rojas M., Salazar-Lezama M.A., Behar S.M. (2012). The Tim3–galectin 9 pathway induces antibacterial activity in human macrophages infected with *Mycobacterium tuberculosis*. J. Immunol..

[13] Jayaraman P., Jacques M.K., Zhu C., Steblenko K.M., Stowell B.L., Madi A., Anderson A.C., Kuchroo V.K., Behar S.M. (2016). Tim3 mediates T cell exhaustion during *Mycobacterium tuberculosis* infection. PLoS Pathog..

[14] Oomizu S., Arikawa T., Niki T., Kadowaki T., Ueno M., Nishi N., Yamauchi A., Hattori T., Masaki T., Hirashima M. (2012). Cell surface galectin-9 expressing th cells regulate Th17 and Foxp3+ treg development by galectin-9 secretion. PLoS ONE.

[15] Qiu Y., Chen J., Liao H., Zhang Y., Wang H., Li S., Luo Y., Fang D., Li G., Zhou B. (2012). Tim-3-expressing CD4^+^ and CD8^+^ T cells in human tuberculosis (TB) exhibit polarized effector memory phenotypes and stronger anti-TB effector functions. PLoS Pathog..

[16] Sada-Ovalle I., Ocana-Guzman R., Perez-Patrigeon S., Chavez-Galan L., Sierra-Madero J., Torre-Bouscoulet L., Addo M.M. (2015). Tim-3 blocking rescue macrophage and T cell function against *Mycobacterium tuberculosis* infection in HIV^+^ patients. J. Int. AIDS Soc..

[17] Chagan-Yasutan H., Ndhlovu L.C., Lacuesta T.L., Kubo T., Leano P.S., Niki T., Oguma S., Morita K., Chew G.M., Barbour J.D. (2013). Galectin-9 plasma levels reflect adverse hematological and immunological features in acute dengue virus infection. J. Clin. Virol..

[18] Dembele B.P., Chagan-Yasutan H., Niki T., Ashino Y., Tangpukdee N., Shinichi E., Krudsood S., Kano S., Hattori T. (2016). Plasma levels of galectin-9 reflect disease severity in malaria infection. Malar. J..

[19] Merani S., Chen W., Elahi S. (2015). The bitter side of sweet: The role of galectin-9 in immunopathogenesis of viral infections. Rev. Med. Virol..

[20] Anderson A.C., Joller N., Kuchroo V.K. (2016). Lag-3, Tim-3, and Tigit: Co-inhibitory receptors with specialized functions in immune regulation. Immunity.

[21] Chagan-Yasutan H., Tsukasaki K., Takahashi Y., Oguma S., Harigae H., Ishii N., Zhang J., Fukumoto M., Hattori T. (2011). Involvement of osteopontin and its signaling molecule CD44 in clinicopathological features of adult T cell leukemia. Leuk. Res..

[22] Zhang J., Yamada O., Kida S., Matsushita Y., Yamaoka S., Chagan-Yasutan H., Hattori T. (2011). Identification of CD44 as a downstream target of noncanonical NF-kappab pathway activated by human T-cell leukemia virus type 1-encoded tax protein. Virology.

[23] Alves C.S., Yakovlev S., Medved L., Konstantopoulos K. (2009). Biomolecular characterization of CD44-fibrin(ogen) binding: Distinct molecular requirements mediate binding of standard and variant isoforms of CD44 to immobilized fibrin (ogen). J. Biol. Chem..

[24] Baaten B.J., Li C.R., Deiro M.F., Lin M.M., Linton P.J., Bradley L.M. (2010). CD44 regulates survival and memory development in T_h_1 cells. Immunity.

[25] Goletti D., Butera O., Bizzoni F., Casetti R., Girardi E., Poccia F. (2006). Region of difference 1 antigen-specific CD4^+^ memory T cells correlate with a favorable outcome of tuberculosis. J. Infect. Dis..

[26] Wu C., Thalhamer T., Franca R.F., Xiao S., Wang C., Hotta C., Zhu C., Hirashima M., Anderson A.C., Kuchroo V.K. (2014). Galectin-9–CD44 interaction enhances stability and function of adaptive regulatory T cells. Immunity.

[27] Wang J., Liu Y., Zhang C.L., Ji B.Y., Zhang L.Z., Shao Y.Z., Jiang S.L., Suzuki Y., Nakajima C., Fan C.L. (2011). Genotypes and characteristics of clustering and drug susceptibility of *Mycobacterium tuberculosis* isolates collected in Heilongjiang province, China. J. Clin. Microbiol..

[28] Chiacchio T., Petruccioli E., Vanini V., Cuzzi G., Pinnetti C., Sampaolesi A., Antinori A., Girardi E., Goletti D. (2014). Polyfunctional T-cells and effector memory phenotype are associated with active TB in HIV-infected patients. J. Infect..

[29] Petruccioli E., Petrone L., Vanini V., Sampaolesi A., Gualano G., Girardi E., Palmieri F., Goletti D. (2013). IFNγ/TNFα specific-cells and effector memory phenotype associate with active tuberculosis. J. Infect..

[30] Brighenti S., Andersson J. (2012). Local immune responses in human tuberculosis: Learning from the site of infection. J. Infect. Dis..

[31] Koguchi Y., Kawakami K., Uezu K., Fukushima K., Kon S., Maeda M., Nakamoto A., Owan I., Kuba M., Kudeken N. (2003). High plasma osteopontin level and its relationship with interleukin-12-mediated type 1 T helper cell response in tuberculosis. Am. J. Respir. Crit. Care Med..

[32] Uede T. (2011). Osteopontin, intrinsic tissue regulator of intractable inflammatory diseases. Pathol. Int..

[33] Inoue M., Shinohara M.L. (2015). Cutting edge: Role of osteopontin and integrin α V in T cell-mediated anti-inflammatory responses in endotoxemia. J. Immunol..

[34] Nau G.J., Chupp G.L., Emile J.F., Jouanguy E., Berman J.S., Casanova J.L., Young R.A. (2000). Osteopontin expression correlates with clinical outcome in patients with mycobacterial infection. Am. J. Pathol..

[35] Nau G.J., Guilfoile P., Chupp G.L., Berman J.S., Kim S.J., Kornfeld H., Young R.A. (1997). A chemoattractant cytokine associated with granulomas in tuberculosis and silicosis. Proc. Natl. Acad. Sci. USA.

[36] Krishnan L., Gurnani K., Dicaire C.J., van Faassen H., Zafer A., Kirschning C.J., Sad S., Sprott G.D. (2007). Rapid clonal expansion and prolonged maintenance of memory CD8^+^ T cells of the effector (CD44^high^ CD62l^low^) and central (CD44^high^ CD62l^high^) phenotype by an archaeosome adjuvant independent of TLR2. J. Immunol..

[37] Gey van Pittius N.C., Warren R.M., van Helden P.D. (2002). ESAT-6 and CFP-10: What is the diagnosis?. Infect. Immunity.

[38] Ridruechai C., Sakurada S., Yanai H., Yamada N., Kantipong P., Piyaworawong S., Dhepakson P., Khusmith S., Keicho N. (2011). Association between circulating full-length osteopontin and IFN-γ with disease status of tuberculosis and response to successful treatment. Southeast Asian J. Trop. Med. Public Health.

[39] Schnittman S.M., Lane H.C., Greenhouse J., Justement J.S., Baseler M., Fauci A.S. (1990). Preferential infection of CD4^+^ memory T cells by human immunodeficiency virus type 1: Evidence for a role in the selective T-cell functional defects observed in infected individuals. Proc. Natl. Acad. Sci. USA.

[40] Groot F., van Capel T.M., Schuitemaker J., Berkhout B., de Jong E.C. (2006). Differential susceptibility of naive, central memory and effector memory T cells to dendritic cell-mediated HIV-1 transmission. Retrovirology.

[41] Chagan-Yasutan H., Saitoh H., Ashino Y., Arikawa T., Hirashima M., Li S., Usuzawa M., Oguma S., EF O.T., Obi C.L. (2009). Persistent elevation of plasma osteopontin levels in hiv patients despite highly active antiretroviral therapy. Tohoku J. Exp. Med..

[42] Li Q., Lifson J.D., Duan L., Schacker T.W., Reilly C., Carlis J., Estes J.D., Haase A.T. (2005). Potential roles of follicular dendritic cell-associated osteopontin in lymphoid follicle pathology and repair and in B cell regulation in HIV-1 and SIV infection. J. Infect. Dis..

[43] Eastwood J.B., Corbishley C.M., Grange J.M. (2001). Tuberculosis and the kidney. J. Am. Soc. Nephrol. JASN.

[44] Berry M.P., Graham C.M., McNab F.W., Xu Z., Bloch S.A., Oni T., Wilkinson K.A., Banchereau R., Skinner J., Wilkinson R.J. (2010). An interferon-inducible neutrophil-driven blood transcriptional signature in human tuberculosis. Nature.

[45] Kaufmann S.H., Lange C., Rao M., Balaji K.N., Lotze M., Schito M., Zumla A.I., Maeurer M. (2014). Progress in tuberculosis vaccine development and host-directed therapies—A state of the art review. Lancet Respir. Med..

[46] Zumla A., Rao M., Dodoo E., Maeurer M. (2016). Potential of immunomodulatory agents as adjunct host-directed therapies for multidrug-resistant tuberculosis. BMC Med..

[47] Dufour J.H., Dziejman M., Liu M.T., Leung J.H., Lane T.E., Luster A.D. (2002). IFN-γ-inducible protein 10 (IP-10; CXCL10)-deficient mice reveal a role for IP-10 in effector T cell generation and trafficking. J. Immunol..

[48] Zhu Y.A., Jia H.Y., Chen J.N., Cui G.Y., Gao H., Wei Y.F., Lu C., Wang L., Uede T., Diao H.Y. (2015). Decreased osteopontin expression as a reliable prognostic indicator of improvement in pulmonary tuberculosis: Impact of the level of interferon-γ-inducible protein 10. Cell. Physiol. Biochem..

[49] Petrone L., Cannas A., Vanini V., Cuzzi G., Aloi F., Nsubuga M., Sserunkuma J., Nazziwa R.A., Jugheli L., Lukindo T. (2016). Blood and urine inducible protein 10 as potential markers of disease activity. Int. J. Tuberc. Lung Dis..

[50] Petrone L., Chiacchio T., Vanini V., Petruccioli E., Cuzzi G., di Giacomo C., Pucci L., Montalbano M., Lionetti R., Testa A. (2014). High urine IP-10 levels associate with chronic HCV infection. J. Infect..

[51] Antonelli A., Ferrari S.M., Corrado A., di Domenicantonio A., Fallahi P. (2015). Autoimmune thyroid disorders. Autoimmun. Rev..

[52] Bifani P.J., Mathema B., Kurepina N.E., Kreiswirth B.N. (2002). Global dissemination of the *Mycobacterium tuberculosis* W-Beijing family strains. Trends Microbiol..

[53] Parwati I., van Crevel R., van Soolingen D. (2010). Possible underlying mechanisms for successful emergence of the *Mycobacterium tuberculosis* Beijing genotype strains. Lancet Infect. Dis..

[54] Zhang J., Mi L., Wang Y., Liu P., Liang H., Huang Y., Lv B., Yuan L. (2012). Genotypes and drug susceptibility of *Mycobacterium tuberculosis* isolates in Shihezi, Xinjiang province, China. BMC Res. Notes.

[55] Kato-Maeda M., Shanley C.A., Ackart D., Jarlsberg L.G., Shang S., Obregon-Henao A., Harton M., Basaraba R.J., Henao-Tamayo M., Barrozo J.C. (2012). Beijing sublineages of *Mycobacterium tuberculosis* differ in pathogenicity in the guinea pig. Clin. Vaccine Immunol..

[56] Szeliga J., Daniel D.S., Yang C.H., Sever-Chroneos Z., Jagannath C., Chroneos Z.C. (2008). Granulocyte-macrophage colony stimulating factor-mediated innate responses in tuberculosis. Tuberculosis.

[57] Who Guidelines on Tuberculosis. http://www.who.int/publications/guidelines/tuberculosis/en/.

[58] Ralph A.P., Ardian M., Wiguna A., Maguire G.P., Becker N.G., Drogumuller G., Wilks M.J., Waramori G., Tjitra E., Sandjaja (2010). A simple, valid, numerical score for grading chest X-ray severity in adult smear-positive pulmonary tuberculosis. Thorax.

[59] Grassinger J., Haylock D.N., Storan M.J., Haines G.O., Williams B., Whitty G.A., Vinson A.R., Be C.L., Li S.H., Sorensen E.S. (2009). Thrombin-cleaved osteopontin regulates hemopoietic stem and progenitor cell functions through interactions with α_9_β_1_ and α_4_β_1_ integrins. Blood.

